# A meta-analysis of relationships between polychlorinated biphenyl exposure and performance across studies of free-ranging tree swallows (*Tachycineta bicolor*)

**DOI:** 10.1098/rsos.150634

**Published:** 2016-04-27

**Authors:** Frances Bonier

**Affiliations:** Department of Biology, Queen's University, Kingston, Ontario, CanadaK7L 3N6

**Keywords:** ecotoxicology, meta-analysis, polychlorinated biphenyls, population-level effects, reproductive success

## Abstract

Habitats worldwide are increasingly being degraded by human activities, with environmental pollution representing a significant threat to species and ecosystems. The presence of persistent organic chemicals, such as polychlorinated biphenyls (PCBs), has generated concern. Captive experiments and field studies have reported some evidence for detrimental effects of PCB exposure, but also significant variation across studies and species. Here, I use a meta-analytical approach to combine findings across 10 studies investigating effects of PCBs on performance (e.g. reproductive success, offspring growth) in free-ranging tree swallows, a common bioindicator species that accumulates high levels of PCBs at some contaminated sites. Contrary to predictions, five complementary analyses revealed no significant negative association between PCB exposure and performance in tree swallows. In fact, in one analysis, increased PCB exposure was associated with improved reproductive success. Possible explanations for these findings include several limitations of field studies, variation in the toxicity of different PCB congener mixtures found across sites included in the analysis, and variation in the degree of tolerance of PCB exposure among species (with high tolerance found in tree swallows). At this point, the available evidence from field studies does not demonstrate negative impacts of PCB exposure on tree swallow performance.

## 1. Introduction

One of the most significant challenges facing organisms worldwide is habitat loss and degradation, often caused in part by environmental pollutants [1–3]. Chemical products and by-products are often intentionally or accidentally discharged into the environment as a result of agriculture, industry and other human endeavours [4,5]. Ecotoxicologists strive to understand the risks posed by these chemicals, and the effects that they have on individuals, populations and ecosystems [6,7]. Yet, our understanding of these impacts is limited for many organisms and many pollutants.

Polychlorinated biphenyls (PCBs) are a widespread and persistent organic pollutant with reported detrimental health effects for vertebrates [8–12], including humans (reviewed in [13,14]). As such, PCBs are of interest to ecotoxicologists, wildlife managers, industry and government agencies. Much of the existing evidence of effects of PCBs has been collected in captive studies. Although the captive setting provides some advantages, such as controlled conditions that allow for experimental manipulations that are not as feasible in a natural environment, captive studies cannot replicate natural environmental exposure to contaminants (e.g. route and temporal profile of exposure, dynamic interactions with other factors such as diet, stress and life history). Thus, perhaps unsurprisingly, empirical evidence for impacts of PCBs from natural systems studied in the field is often inconsistent, both with predictions based on controlled captive studies [9,11], and across species [15,16] and field sites (cf. [17,18]). In order to better understand and predict how PCBs will influence a population, species or ecosystem of interest, we need a firmer grasp on the ways in which PCBs impact free-ranging animals in a natural setting.

Recognizing this need, ecotoxicologists often compare contaminant-exposed and reference populations of bioindicator species (i.e. species that can be used as biological proxies for other taxonomically similar species). These field studies address some of the limitations of captive studies—they are conducted in a natural setting, and involve observation of biologically relevant performance metrics (e.g. behaviour, breeding success, offspring development, recruitment, survival) and their relationships with estimates of natural exposure to contaminants. Such studies have provided some correlative evidence of reduced performance associated with exposure to PCBs in free-ranging animals (e.g. [19–21]). However, like captive studies, some field studies also suffer from limitations. For example, the scope of these studies is typically limited—involving comparisons of a relatively small number of individuals from contaminated areas to individuals from reference sites that are predicted to experience lower levels of PCB exposure (e.g. [22,23]). Ideal control populations are rarely sampled, and perhaps not available [24]; and reference sites can differ in a number of habitat features, not only in PCB contamination levels [23]. These field studies are almost always correlative, and the small number of populations considered in many field studies limits statistical power to test hypotheses regarding PCB effects. In an ideal ecotoxicology field study, we would compare measures of performance of individuals found in several sites contaminated with only the pollutant of interest (in this case, PCBs) with performance of individuals found in several pristine, uncontaminated sites that are similar to the contaminated sites in every way, apart from the presence of the pollutant. In reality, such comparisons are rarely possible for a number of reasons. First, different contaminants and different congeners of contaminants with varying degrees of toxicity can co-occur in a given site, and individuals of some species can readily move among sites with varying levels and types of contamination, and so isolating effects of one contaminant of interest in a field setting can be difficult. Second, completely pristine sites that could serve as perfect controls (i.e. identical in every way other than contamination with the pollutant of interest) are simply not available. And finally, several of the metrics of performance that might be most valuable—for example, survival and recruitment—are very difficult to accurately estimate in free-ranging organisms. Thus, at present, evaluating the evidence from the growing body of wildlife ecotoxicology studies regarding the impacts of PCBs is challenging.

Meta-analytical methods offer a potentially robust approach to address this challenge and investigate the overall weight of current evidence to seek broad patterns of relationships between PCBs and performance [24–27]. Meta-analyses allow integration of findings across multiple studies and multiple performance metrics, and thus can provide robust and broad tests of hypotheses [26,27]. Meta-analyses are not influenced by the findings of individual studies, but instead combine data across studies into one analysis to investigate broader patterns. As such, the effect of limitations of individual studies (as described above) can be reduced by combining findings across studies that probably vary in the relative influence of these limitations. Finally, the data from individual studies can be weighted to allow increased influence of more precise estimates relative to more coarse estimates in the meta-analysis (e.g. by weighting analyses by the inverse of variance of estimates or by study sample size) [28]. Despite the many strengths of meta-analyses for synthesis of findings across studies and detection of broad patterns that cannot be revealed in single studies, they too are associated with limitations, the most significant of which is limitation of the data on which they rely. Meta-analysis is most appropriate for addressing research questions with data available from a relatively large number of studies, and the robustness of findings from meta-analyses increases with the number of studies and the sample size of those studies [29].

The tree swallow (*Tachycineta bicolor*) is an ideal candidate for a meta-analytical approach, because it has been the subject of numerous ecotoxicological studies across North America. Tree swallows have long been considered a preferred bioindicator, or sentinel, species for PCB and other contaminant studies for a number of reasons, including their amenability to intensive study in part because of their willingness to nest in artificial nest-boxes, their close ties to habitats that are the sites of PCB contamination, and their reliance during the breeding season on aerial insects, often with benthic aquatic larvae, for feeding themselves and their offspring [30]. Several studies report that tree swallows accumulate PCBs during the breeding season at contaminated sites, presumably through feeding on contaminated insects [31–33]. Some of the reported PCB concentrations in tree swallow eggs, nestlings and adults are among the highest reported PCB exposure levels in nature (some examples of estimated PCB exposure levels in tree swallows can be found in table 1). For example, McCarty & Secord [21] report tree swallow egg total PCB concentrations as high as 29 500 ng g^−1^—a concentration an order of magnitude greater than the LD50 for avian embryos reported across several other bird species (reviewed in [15]). In the field, a number of studies have been aimed at detecting impacts of PCBs on tree swallows by comparing performance estimates (e.g. breeding success, nestling growth) of individuals found in contaminated and reference study sites, providing data both on these metrics and on estimates of PCB exposure derived from tree swallow tissue samples collected at the same sites.

**Table 1.** A summary of the studies included in the meta-analyses.

reference	location	PCBs in the lowest and highest level sites^a^	performance metrics^b^
[22]	Ontario, Canada	286.7 2541^c^	first egg date, clutch size, hatching success, fledging success
[34]	Kalamazoo River, MI, USA	460 3100	clutch size, egg mass, hatching success, fledging success, nestling mass, nestling body length, nestling tarsus, nestling wing chord
[35]	Housatonic River, MA, USA	1015 34 957	hatching success
(C Bishop 2014, personal communication and Environment Canada; [36])	Great Lakes and St Lawrence River Basin, USA and Canada	45.8 5469	clutch size, hatching success, fledging success, nestling mass
[37]	Fraser River Drainage, British Columbia, Canada	0.1 31.6	clutch size, hatching date, fledging success, nestling mass, nestling wing chord
[38]	Fox River Drainage, WI, USA	80 3770	clutch size, embryo mortality, hatching success
[39]	Hudson River Valley, NY, USA	377 or 5250^d^ 55 800	nest abandonment, first egg date, clutch size, egg mass, hatching success, fledging success, nestling mass, nestling wing chord
[40]	Hudson River Valley, NY, USA	721 62 200	number of feathers in nest, nest mass
[41]	New Bedford Harbor, MA, and Fox Hill, RI, USA	26 16 800	clutch size, hatching success
[42]	Lake Calumet Region, IL, USA	383.9 618.6	first egg date, clutch size, hatching success, fledging success, nestling mass

a Concentrations of total PCBs measured in tree swallow nestling tissue, except where noted, and reported in nanogram per gram. Some of these values are study site averages calculated from reported annual values for more than 1 year of study.

b Descriptions of all of the performance metrics extracted from each study and included in the analyses.

c Values are for total PCB concentrations in tree swallow eggs, as nestling tissue concentrations were not reported.

d Some performance metrics were not estimated for the lowest PCB site, so the next lowest site was used for those comparisons.

Despite the unquestionable exposure of tree swallows to PCBs and several investigations into the impacts of PCBs on this species, we currently lack clear, consistent evidence for impacts of PCBs on performance in tree swallows. For example, previous field studies report negative [18,34,35], positive [18] and non-significant associations [17,36–38] between estimates of PCB exposure and survival and reproductive success in tree swallows. To evaluate the available evidence, I compiled data from the ecotoxicology literature on PCBs in free-ranging tree swallows and conducted a meta-analysis aimed at testing the hypothesis that PCB exposure negatively impacts performance in these birds.

## 2. Material and methods

### 2.1. Data compilation

I identified relevant sources for the meta-analysis from searches of the published literature and publicly available reports (e.g. environmental consulting and government agency reports) using the search engines Web of Science and Google Scholar with the keywords tree swallow and PCBs. Some additional data that could not be extracted from one publication [36] were acquired through personal communication with the study author (C Bishop 2014, personal communication). I included data based on the following criteria: each paper must report (i) study site level estimates of tree swallow exposure to PCBs (e.g. mean whole-body tissue PCB concentrations in nestling swallows at a particular study site), (ii) study site level estimates of mean performance (e.g. morphology of nestlings, breeding success and breeding behaviour), (iii) sample size used to calculate performance estimates and (iv) the two types of data (PCBs and performance) must have been estimated during the same study period. In addition to the obvious exclusions, these criteria led to exclusion of studies for which study site level mean values for PCBs or performance were not reported, even though they were in some cases included in the analyses in the study (e.g. [18,43,44]). In each of these cases, I contacted authors to request their unpublished data, but, for various reasons, the data were not provided. The resulting dataset included PCB exposure estimates, performance estimates and sample sizes from 10 studies (table 1; electronic supplementary material, figures S1–S9; the data supporting this article has been uploaded as part of the electronic supplementary material). Ideally, for a study of impacts of PCBs, data on all performance metrics likely to impact population demography would be included. However, none of the studies meeting the selection criteria included estimates of survival. Thus, the meta-analysis is limited to investigating effects on adult tree swallow reproductive performance, behaviour and nestling growth.

### 2.2. Performance metrics

I included all reported estimates of performance in tree swallows that could be linked to predicted fitness or demographic effects, including data on fecundity, breeding success, breeding behaviour and nestling growth, and where the direction of improved performance could readily be inferred. For example, greater breeding success is directly linked to increased reproductive fitness and therefore higher values for this metric can be inferred to indicate improved performance. In cases where performance metrics were presented separately for multiple sequential years of study, I calculated a mean performance metric for each site within the individual study using the reported annual means and sample sizes (to determine one integrated study site-level estimate of performance and avoid issues of repeated measurement of the same metric within study sites across years).

For all of the statistical models described below, the dependent variable was a response ratio of the performance metrics [25]. I calculated these response ratios for each study in one of two different ways, depending on the metric type. For metrics where a higher value indicates superior performance (e.g. proportion of clutches that hatched), I calculated the response ratio by dividing the mean performance estimate for tree swallows sampled in the study site with the greatest level of PCB exposure by the performance estimate for swallows from the site with the lowest level of PCB exposure (usually reported as the study's reference site). For example, if the mean nest success in the reference site was 3.6 fledglings, and the mean nest success in the most contaminated site was 3.1 fledglings, the nest success response ratio would be 3.1/3.6 = 0.86. For metrics where a lower value indicates superior performance (e.g. proportion of eggs containing dead embryos), I did the converse, dividing the value reported for the performance metric for tree swallows from the study site with the lowest level of PCB exposure by the performance estimate for swallows from the study site with the greatest level of PCB exposure. In this way, each study was assigned one response ratio for each reported performance metric scaled against performance of swallows from the lowest PCB level study site within that same study. Response ratio values less than one indicate poorer performance and values greater than one indicate superior performance by individuals from higher PCB sites, relative to individuals from the lowest PCB exposure site. These standardized response ratios calculated for varied performance metrics (e.g. breeding productivity or nestling growth) that were estimated using diverse approaches (e.g. hatching success, fledging success or clutch size; and nestling wing length, body mass or tarsus length) allow for comparison of tree swallow performance both within and across studies in one meta-analysis. However, because this method of calculation of response ratios excludes study sites with intermediate PCB exposure, I also conducted supplemental analyses using different methods of calculation that allowed inclusion of all of the sites within a study. Results of these supplemental analyses support similar interpretations as the findings reported below (see the electronic supplementary material).

### 2.3. Statistical analyses

All statistical analyses were conducted using the program R (version 3.0.2, R Core Team 2013). To test the hypothesis that increased exposure to PCBs impacts performance in tree swallows, I conducted several complementary analyses. First, I used generalized linear mixed effects models (GLMMs) using the function *lmer* from the R package *lme4* and fitted using maximum likelihood (*REML* *=* *false*) to allow model selection (see below). In these models, the natural log-transformed performance response ratio was the dependent variable (as recommended in [25]), and the difference in PCB exposure level (based on reported whole egg or nestling tissue concentrations) between the highest and lowest PCB exposure site within the study was a fixed effect (log-transformed to better fit a normal distribution). This difference in PCB exposure allowed for analysis of the relationship between tree swallow performance and the estimated level of exposure at the high-PCB site relative to exposure in the low-PCB site. I also included the log-transformed PCB exposure level of the low-PCB site (i.e. the reference site) as a fixed effect, along with an interaction between this PCB level and the difference in PCB exposure (PCB low × PCB difference). This interaction term allowed for the possibility that PCBs have a threshold of exposure, and performance is not compromised in a linear manner. Under this scenario, tree swallows in reference sites with relatively high levels of contamination might not be predicted to differ in performance from tree swallows in more contaminated sites, whereas tree swallows in reference sites with lower PCB exposure would be predicted to perform better than tree swallows in more contaminated sites. I also included a category for each performance metric as a fixed effect, as well as an interaction term (category × PCB difference). This category classified all performance metrics as either estimates of breeding performance (e.g. clutch size, hatching success) or nestling morphology (e.g. nestling body mass, tarsus length). Inclusion of the interaction term allowed for testing the hypothesis that PCB exposure does not influence both types of performance metrics similarly. Study identity was included as a random effect, to account for non-independence of multiple performance metrics reported within an individual study. I compared models with all combinations of the main effects and the interaction terms specified above, as well as a null model (containing only the random effect) using the function dredge in R (package *MuMln*) and comparing Akaike's Information Criterion corrected for small sample size (AICc) to identify the best-fit model [45]. For these analyses, I report both frequentist and information theoretic statistics in the results [46]. I also report marginal and conditional *R*^2^-values to describe model fit, with the marginal value reflecting model fit only considering the fixed effects, and the conditional value reflecting the random and fixed effects [47]. Most studies did not report variance or confidence intervals for their performance metric estimates, so I conducted analyses weighted by the combined sample size of the two study sites used to calculate each response ratio. This approach allows for greater influence of studies with larger sample sizes and presumably more precise estimates of performance metrics [26]. I also present results from unweighted analyses, for comparison. These primary analyses included a total of 48 response ratios based on estimates of tree swallow performance from 10 studies and 20 sites (one high-PCB and one low-PCB site per study). If increased exposure to PCBs negatively impacts performance in tree swallows, higher levels of PCB exposure should be associated with lower response ratios in these analyses.

I conducted a second, complementary analysis on a subset of the data, only considering fledging success (six studies), or hatching success, for those studies that did not report fledging success (three studies). One of the 10 original studies did not report either metric, and so was excluded from this analysis. This resulted in a dataset that included only one response ratio per study (relative fledging or hatching success), based on the performance metrics that provide the most direct estimates of reproductive success available in these studies. This supplemental analysis excludes metrics that were retained in the primary analysis that might not have clear direct impacts on population status (e.g. nest construction, nestling morphology), and so might be of limited importance in determining population-level impacts of PCBs. In this case, I assessed the relationship between PCB exposure and performance with a standard least-squares regression, with the natural log-transformed reproductive success ratio as the dependent variable and the log-transformed difference in PCB exposure level as the independent variable. If increased exposure to PCBs negatively impacts performance in tree swallows, increasing PCB exposure should be associated with a reduction in reproductive performance ratios in these analyses.

Third, I conducted a simple one-sample *t*-test of the mean of the response ratios for each study to determine whether those values differed from one (*N* = 10 studies, with one mean response ratio per study). A benefit of this relatively simple analysis, as compared to the above two analyses, is that it does not rely on the existence of a linear relationship between PCB exposure and performance. By contrast, this analysis simply looks for an overall reduction in performance for tree swallows in more contaminated sites relative to less contaminated sites, independent of the level of contamination. If PCBs negatively impact performance, the mean of the performance ratios should be less than one.

## 3. Results

Measures of PCB exposure (PCB level at the reference site, difference in PCBs between the highest and lowest level sites, and their interaction) were unrelated to tree swallow performance in the first analysis. The best-fit model was the null model, including only the random effect of study identity (table 2). The inclusion of the difference in PCB levels between sites did not improve the model's ability to explain tree swallow performance ratios, and significantly reduced likelihood of this model relative to the null, as indicated by a 2.22 increase in AICc [45]. In this model, which included PCBs but did not retain the performance metric category, PCBs were not significantly correlated with performance ratios (marginal *R*^2^ < 0.001, conditional *R*^2^ = 0.003, *β* = 0.02, *p* = 0.69). The top-ranked model containing the PCB level in the site with the lowest PCB exposure level had an AICc score only 0.24 greater than the score for the null model, indicating similar support for these two models. In this model, PCBs at the reference site were not significantly correlated with performance ratios (marginal *R*^2^ = 0.003, conditional *R*^2^ = 0.006, *β* = 0.06, *p* = 0.14). The top-ranked model including any interaction term had an AICc score 3.61 greater than the score for the null model, so I only report results for the null model and five main effect models ranked within 2.7 AICc units of the null (table 2). Analyses run without weighting by sample size yield similar results. None of the results from any of these models provide support for the hypothesis that PCBs negatively impact tree swallow performance.

**Table 2.** Comparison of generalized linear mixed effects models (GLMMs) used to explain variation in performance ratios^a^ in tree swallows, analyses run both with (above) and without (below) weighting by sample size.

fixed effects^b^	ΔAICc	*R*^2^-value^c^	estimate^d^	*p*-value
null model	0.0	0, 0.004	—	—
	0.0	0, 0.106		
PCB exposure (reference site)^e^	0.2	0.003, 0.006	0.06 ± 0.04	0.14
	0.7	0.04, 0.06	0.03 ± 0.02	0.18
performance category^f^	2.0	<0.001, 0.004	−0.05 ± 0.07	0.54
	2.1	0.006, 0.100	−0.02 ± 0.04	0.58
difference in PCB exposure^g^	2.2	<0.001, 0.004	0.02 ± 0.04	0.69
	1.1	0.04, 0.06	0.03 ± 0.02	0.18
performance category			−0.04 ± 0.07	0.54
	2.5	0.003, 0.006	−0.02 ± 0.04	0.56
PCB exposure (reference site)	3.0	0.05, 0.12	0.06 ± 0.04	0.15
			0.02 ± 0.02	0.21
difference in PCB exposure			−0.02 ± 0.05	0.67
	2.6	0.003, 0.006	0.01 ± 0.03	0.24
PCB exposure (reference site)	3.1	0.05, 0.10	0.07 ± 0.04	0.15
			0.02 ± 0.02	0.46

a Performance ratios are calculated as described in the main text—either the performance metric for the highest PCB site divided by the performance metric for the lowest PCB site within the same study (for metrics where higher values indicate superior performance), or the converse (lowest PCB performance /highest PCB performance, for metrics where lower values indicate superior performance). Performance ratios were natural log-transformed for analysis.

b All models included study identity as a random effect.

c Both marginal (left) and conditional (right) *R*^2^-values are presented. Marginal *R*^2^ estimates the variation in performance explained by the fixed effects alone, whereas the conditional *R*^2^ estimates the variation explained by the fixed and random effects in combination.

d Effect estimates presented ±s.e.

e PCB exposure as estimated by tissue concentrations of PCBs in nestling or embryo tree swallows sampled in the lowest PCB exposure site within the study, and log-transformed to better fit a normal distribution.

f A categorical fixed effect identifying the performance ratio metric as either breeding performance (e.g. reproductive success) or nestling morphology (e.g. nestling body mass).

g PCB exposure as estimated by tissue concentrations of PCBs in nestling or embryo tree swallows, calculated for these analyses as the difference between the highest PCB exposure site's PCB level and the lowest PCB exposure site's PCB level, and log-transformed to better fit a normal distribution.

**Figure 1.**
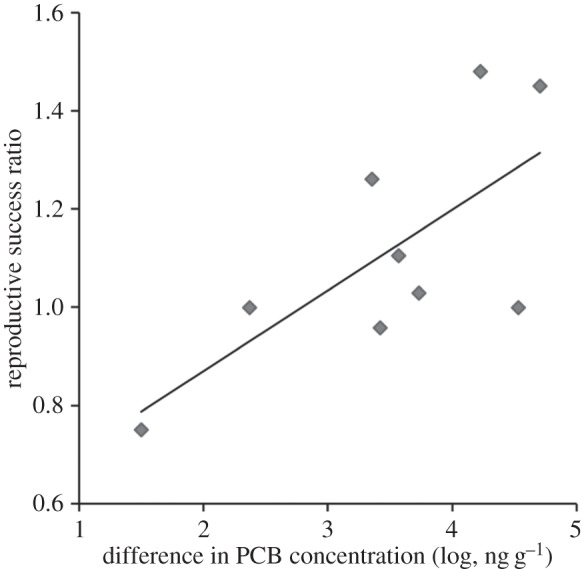
Relative reproductive success of tree swallows increases with PCB exposure (least-squares regression: *N* = 9 studies, *R*^2^ = 0.54, *β* = 0.15, *p* = 0.02). Reproductive success ratios (*y*-axis) are calculated as the mean reproductive success (hatching or fledging success) of tree swallows sampled in the study site with the greatest level of PCB exposure divided by the mean reproductive success of tree swallows from the site with the lowest level of PCB exposure within a given study. Values greater than one indicate greater reproductive success in the higher PCB site. PCB differences (*x*-axis, presented log-transformed) are calculated as the difference between the mean PCB exposure estimate (from nestling or embryo tissue) of the highest and lowest PCB sites within a study.

In the second analysis, the relative reproductive success (response ratio of hatching or fledging success) of individuals sampled in the study site with the highest level of PCB exposure increased with the difference in PCB levels between this high-PCB site and the reference site (figure 1; standard least-squares regression, *N* = 9 studies, *R*^2^ = 0.54, *β* = 0.15, *p* = 0.02). In other words, tree swallows in highly contaminated sites (as compared to the same study's reference site) had greater relative reproductive success than swallows in sites with more similar PCB levels between the high- and low-PCB sites. This finding is opposite to predictions of the hypothesis that PCBs negatively impact performance in tree swallows.

**Figure 2.**
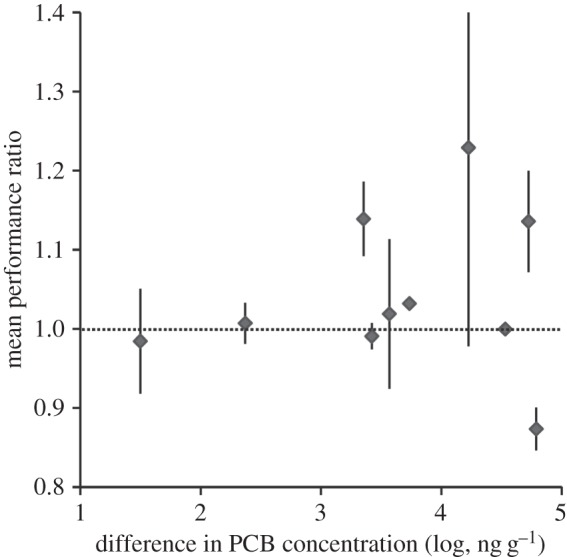
Mean performance of tree swallows does not differ between sites with the greatest and lowest levels of PCB exposure (one-sample *t*-test comparing observed values to 1: *N* = 10 studies, *t* = 1.29, *p* = 0.23). Mean performance ratios (*y*-axis, ±s.e.) are calculated as the mean of all of the reported performance metrics within a study for the site with the greatest level of PCB exposure relative to the site with the lowest level of exposure. Values greater than one (dashed line) indicate superior performance in the high-PCB site relative to the low-PCB site. These metrics are presented here plotted against the difference in PCB exposure of the highest and lowest PCB sites (*x*-axis, presented log-transformed).

Finally, the last analysis shows similar overall performance across all metrics between tree swallows sampled in the highest and lowest PCB exposure site within each study, as indicated by mean performance response ratios across studies that did not differ from one (figure 2; overall mean response ratio = 1.04, *N* = 10 studies, one-sample *t*-test: *t* = 1.29, *p* = 0.23). Again, this finding does not follow the predictions of the hypothesis that PCBs negatively impact performance in tree swallows.

## 4. Discussion

Using several complementary meta-analytical approaches to assess findings across field studies on PCBs and tree swallows, I found no evidence of detrimental effects of PCBs on performance. When considering a suite of metrics, the difference in PCB exposure between the contaminated and reference sites did not predict tree swallow performance (table 2). In a complementary analysis of a subset of the data only including the most direct estimates of reproductive performance (fledging or hatching success), tree swallow performance actually increased with relative level of PCB exposure (figure 1). Finally, in a simple test of mean performance across all metrics, tree swallows from study sites with the highest PCB exposure had similar performance as swallows from sites with the lowest PCB exposure (figure 2).

In contrast to the findings reported here, prior experimental work with captive animals (e.g. mice, chickens, American kestrels and common terns) reports that PCBs can compromise performance, often at exposures much lower than those experienced by the swallows in this dataset (e.g. [9–12,48]). How can we resolve these apparently contradictory findings? One plausible explanation is that correlative field studies might be insufficient for detecting detrimental impacts of PCBs. Several factors combine to influence an individual's performance, including age, experience, habitat quality and mate quality. Study sites sampled in the field studies included in this meta-analysis undoubtedly differ in many of these factors, and these differences might obscure the influence of PCBs. For example, reference sites, despite having lower levels of PCBs, might be located in lower quality habitat, confounding comparisons because they are more likely to be occupied by younger or lower quality birds that are constrained to having lower reproductive success or poorer performance than individuals that settle in sites with greater levels of contamination. Additionally, both reference and contaminated sites can be contaminated with other pollutants, which might influence Tree Swallow performance (e.g. [22,37,38]). Unfortunately, data on all of the factors that influence study site habitat quality are generally not available and cannot easily be controlled. However, in a meta-analysis such as this, the influence of these unmeasured factors should be minimized through the increased power to detect effects of PCBs gained by combining findings across studies and sites.

Variation in the toxicity of specific PCB mixtures might also contribute to these findings. PCB congeners differ in their toxicity [49,50], and the ratios of congeners undoubtedly differ among sites included in this meta-analysis (although congener profiles were not available for most studies, and so inclusion of toxic equivalency factors in analyses was not possible). Thus, we might expect differing degrees of effects on performance that are more directly related to toxicity of specific PCB congeners than to total PCB exposure. However, captive toxicity tests using the same mixture and concentrations of PCB congeners as is found in at least one of the sites of the tree swallow studies included in this meta-analysis report significant effects on hatchability and nestling development at even relatively low levels of exposure [48]. These levels of PCBs that had a measurable impact in captive studies are lower than the levels that apparently fail to impact reproductive success in free-ranging tree swallows. However, this previous study was conducted on domestic chickens, which have experienced a very different, artificial selection regime than any wild bird species, and are known to be highly sensitive to several toxins, including PCBs [51]. Furthermore, even if the degree of toxicity of different congener mixtures varies across studies, we would still predict a general negative impact of PCBs on performance, particularly with increasing levels of exposure. By contrast, we found evidence for the opposite pattern, with increasing levels of exposure associated with improved reproductive performance in an analysis of the most direct metrics of reproductive success available in the dataset (figure 2).

An additional, and non-exclusive explanation of the contrast between the results presented here and findings of captive experiments is that some animals, like the tree swallow, might tolerate greater levels of PCB exposure than those species that have been the focus of captive experimental studies. Physiological tolerance of pollutants could be reduced during the lengthy history of artificial selection that has produced the captive strains of animals that are most often used in experimental studies (e.g. chicken, Japanese quail, laboratory strains of mice). Indeed, significant among-species variation in toxicity is commonly reported (e.g. [11,52,53]), and a genetic basis for among-species differences in toxicity of PCBs has been described in birds [15].

Finally, detrimental effects of PCB exposure might not be evident in the performance metrics included in this meta-analysis. For PCBs to negatively impact populations, they must compromise lifetime reproductive success. Here, I find no evidence for detrimental impacts on estimates of reproductive success; however, the metrics available are all fairly short term, involving measurement of hatching or fledging success. PCBs might influence recruitment of young birds, through compromised survival during their first year and/or through reduced fecundity of those birds. Effects on survival of first-year birds would be very difficult to detect in this species, and would require sampling of a large number of nestlings and intensive recapture efforts, as most populations normally experience very low recruitment rates (0.8–12%; [54]). However, studies investigating individual-level associations between PCB exposure and survival of adult tree swallows report contrasting evidence, with one study reporting some evidence of detrimental effects on survival [18], and one study finding no evidence of effects on survival [17]. It should be noted that the study that reported some evidence of a negative influence of PCB exposure on survival [18] had higher estimates of total PCB exposure (but similar toxic equivalents (TEQs)) than the other study [17], which failed to find negative effects on survival. Custer *et al*. [18] also reported that PCB content of eggs of females that survived and returned in the following year to the study site were similar to or even higher than PCB content of eggs laid by females that failed to return. The authors attribute this unexpected finding to the possibility that females in better condition (and thus more likely to survive to the following year) might have more lipid stores, and therefore deposit more PCB-contaminated lipid into their eggs [18]. In this case, the female's body condition and lipid stores might directly influence survival and also the amount of PCB deposited in the egg. This possibility, while plausible, also suggests that using egg PCB content as an estimate of adult PCB exposure might be unwise, given that it could be confounded by the adult female's body lipid stores.

The positive relationship between tree swallow reproductive performance and PCB exposure described here (table 2 and figure 2) is more challenging to understand. None of the caveats and limitations described above can explain this finding. Even if (i) tree swallows are more tolerant of PCBs than some captive-bred and domesticated species, (ii) reference sites sometimes attract lower quality birds or are otherwise less than perfect, (iii) toxicities of PCB congener mixtures differ among studies and/or (iv) impacts of PCBs are not detected with the metrics and analyses used here, we would not predict this positive relationship. Under the second explanation (reference sites might attract lower quality birds), we would predict higher fitness in birds from contaminated sites, but we would not predict the finding of an increase in relative fitness with the level of contamination. PCBs are unlikely to directly increase reproductive success. One plausible explanation is that researchers are constrained to selecting reference sites that are more different and/or distant for comparison to the most highly contaminated sites, because more appropriate reference sites are not available due to the geographical scale of contamination at the most heavily contaminated sites. Alternatively, heavily contaminated sites might be more protected from other disturbances and sources of habitat degradation that can compromise Tree Swallow performance (development, exploitation, recreational use, etc.) than reference sites, making them more suitable and productive habitat for tree swallows. These possibilities could only explain this finding if there is some systematic bias whereby reference sites are routinely lower quality sites than highly contaminated sites. If that were the case, then all of the existing field comparisons of tree swallow performance in contaminated and reference sites would be called into question. Finally, across all field studies, individuals breeding in contaminated sites might represent a non-random sample comprising only those individuals that are unusually tolerant and able to survive and reproduce in the face of strong selection imposed by the contaminant. If the most highly contaminated sites exert the strongest selection, we might predict a positive association between level of contamination and reproductive success, reflecting the high-quality or other positive attributes of the individuals that can persist and breed in these sites. At present, this result is based on findings from a limited amount of data (*N* = 9 studies), and so we must await further work to determine if the relationship persists, and, if it does, to reveal its cause.

Future work investigating possible effects of PCBs on this sentinel species should involve more careful selection of reference sites and increased sampling across multiple sites of varying degrees of contamination. Overall, these field studies should avoid confounds where possible, particularly in the selection of reference sites [24]. Additionally, further analyses of individual-level exposure and performance would be valuable [30]. For example, investigating individual-level patterns could involve measuring PCB concentration in an egg and subsequent survival (hatching and fledging) of the embryos and nestlings from the remaining eggs from the same clutch. Relatively new methods for measuring PCB concentrations in small volumes of blood might provide a useful tool, allowing for non-lethal sampling and estimation of PCB exposure, and subsequent tracking of reproductive success and survival of the same individuals [55]. An individual-level approach could reduce error associated with variation among individuals in exposure that cannot be accounted for in study site level analyses, such as those included in this meta-analysis, where mean site-level exposure estimates based on tissue samples are assumed to reflect individual exposure. Tree swallows and other migratory birds typically spend most of their lives away from the breeding site, on migratory routes and wintering grounds, and so exposure to contaminants is likely to vary significantly among individuals, even within breeding sites. Therefore, studies looking directly for relationships between exposure and performance within individuals would allow for a more refined analysis of the consequences of PCB exposure. These individual-level results could be scaled up to predict demographic impacts in order to understand how PCBs affect population status. Experimental studies could be used to test the hypothesis that contaminants impose selection that favours persistence of high-quality individuals in contaminated sites. Finally, although captive work on tree swallows is not feasible, further experimental studies of wild-derived species could provide toxicity data for use in a comparative framework to investigate the degree to which species differ in their sensitivity to PCBs.

## Supplementary Material

2. All data (excel file)

## Supplementary Material

1. Supplemental analyses and figures (word doc)

## Data accessibility

The data used for the analyses are available as electronic supplementary material.
