# Supplementary material for: A meta-analysis of relationships between polychlorinated biphenyl exposure and performance across studies of free-ranging tree swallows (Tachycineta bicolor)
Source: R Soc Open Sci. 2016 Apr 27;3(4):150634. doi: 10.1098/rsos.150634 (PMC4852628; doi:10.1098/rsos.150634)
Supplement: 1. Supplemental analyses and figures (word doc) [file rsos150634supp2.doc]

Title:

**Online Supplemental Material**

A meta-analysis of relationships between PCB exposure and performance across studies of free-ranging Tree Swallows (*Tachycineta bicolor*)

Frances Bonier, Queen's University, Kingston, Ontario, Canada

As a supplement to the analyses presented in the main text, I conducted additional analyses that allowed inclusion of all of the study sites sampled within each study. Methods and results for these analyses are provided below. All of the data on performance estimates and PCB exposure that were used in these analyses are plotted in Figures S1 through S9. Note that the analyses in the main text compared only the highest and lowest PCB exposure sites within each study.

**Methods**

*Response Ratios*

For these supplemental analyses, I calculated response ratios for each study site (instead of just the highest compared to lowest PCB site, as in the main text) within each study in one of two different ways, depending on the metric type. For metrics where a higher value would be interpreted as superior performance (e.g., proportion of clutches that hatched), I calculated the response ratio by dividing the performance estimate for a given study site by the highest value for that performance metric reported within the same study. And for metrics where a lower value would be interpreted as superior performance (e.g., proportion of eggs containing dead embryos), I divided the lowest value reported for the performance metric within a given study by the performance metric for the study site of interest. In this way, each site within a study was assigned a response ratio for each performance metric scaled against the best-performing site within that same study (which would be assigned a response ratio of one). This approach allows for comparison of response ratios calculated for different performance metrics both within and across studies.

*Statistical analyses*

All statistical analyses were conducted using the program R (R Core Team 2013). Response ratios yielded performance endpoint estimates between zero and one, with an excess of values equal to one and a strong skew towards zero. To match statistical models with this distribution, I transformed the response ratios to reverse the skew direction and transform all data to integers (resulting in an excess of zeroes with a right-hand skew, using the following equation: integer[(1-response_ratio)*100]), and then used generalized linear mixed-effects models with zero-inflated distributions to analyze the data. All saturated models included the site-specific estimate of PCB exposure, as estimated by nestling PCB concentrations. One study that was included in the main text was thus excluded from these analyses, as it did not report nestling PCB concentrations (Gilchrist et al. 2014). I transformed PCB levels prior to analysis so that their distribution was closer to a Gaussian distribution (using the equation: *ln[total PCB in nestlings+1]*); I then centered the data (using the equation: *cen.PCB=[logPCB-mean(logPCB)]*) following Bolker et al. (2009). Mixed-effects models were necessary because studies often reported multiple performance endpoints on the same individuals, and thus these measures were not independent. I used the statistical package glmmADMB (Skaug et al. 2013) in R to test the hypothesis that levels of PCB contamination influenced performance in Tree Swallows, using two different tests.

The first test used glmmADMB, with the transformed performance endpoint response ratios as the independent variable, transformed nestling PCB concentrations and category (category of performance metrics, either breeding or nestling morphology) as fixed effects in a saturated model, and study as a random effect, following Bolker et al. (2012ab). I compared models with the following distributions: poisson, negative binomial where (variance = µ(1+ µ/k)), and negative binomial where (variance = Φ µ), and also compared models that specify the presence or absence of zero inflation. I assessed the performance of these different models using Akaike Information Criteria values that control for small sample size (AICc), where smaller values indicated better model performance. Once I had identified the best distribution for the model, I ran all combinations of fixed effects (saturated, additive, PCB only, category only, and a null model), and compared the performance of these models using AICc values.

The second test used hurdle models in glmmADMB, with the same independent, fixed, and random factors as described above, following Bolker et al. (2012b). Hurdle models partition zero-inflated analyses into one test involving zeros and non-zeros (binomial test), and a second test only on non-zero values (count test). This approach can be powerful if PCBs influence performance endpoints in different ways. I partitioned the dataset following Bolker et al. (2012b) with a binomial distribution for the bivariate zero and non-zero (=1), and the "truncnbinom1" family for the values above zero. I ran analyses on the data as described for the previous glmmADMB analysis, comparing fixed effects using AICc values.

**Results**

The best-performing model using the glmmADMB approach had a negative binomial distribution (variance = Φ µ) and accounted for zero inflation (ΔAICc>2 for all other models). In the first analysis (negative binomial, zero-inflated), the best-performing model had only performance metric category represented (Table S1), indicating that response ratios differ for breeding versus nestling morphology metrics. The closest model with PCB level was the additive model (PCB + category), and had a ΔAICc of just over 2 (Table S1), indicating significantly worse fit relative to the model with category alone. The best performing hurdle models was the null model for the binomial (zero, non-zero) component, and the model only including category for the non-zero (count) components (Table S1). The closest-performing model with PCB level included had a ΔAICc of 1.1 (binomial) and 1.9 (count) relative to the null model (Table S1). For models with PCB levels as the sole fixed factors, the estimate of PCB effects on performance endpoints was negative, but in all cases with standard errors of those estimates overlapping zero and *p*-values > 0.3 (note that negative values are indicated here as positive because we inverted the response ratios prior to analysis: *ß* = 0.0158 ± 0.048SE, *p* = 0.74, negative binomial, zero-inflated model; *ß* = 0.071 ± 0.075SE, *p* = 0.35, hurdle model, binomial component; *ß* = 0.0049±0.035SE, *p* = 0.89, hurdle model, non-zero component).

Overall, these findings are qualitatively similar to the results presented in the main text. No significant evidence for an association between PCB exposure and performance was detected.

**References**

Bolker, BM, ME Brooks, CJ Clark, SW Geange, JR Poulsen, MHH Stevens, and J-SS White. 2009. Generalized linear mixed models: a practical guide for ecology and evolution. *Trends in Ecology and Evolution* 24:127-135.

Bolker, BM, ME Brooks, B Gardner, C Lennert, and M Minami. 2012a. Owls example: a zero-inflated, generalized linear mixed model for count data. Online at: *http://*ms.mcmaster.ca/~bolker/misc/nceas_nonlinear/owls/owls.pdf*‎*

Bolker, BM, H Skaug, A Magnusson, and A Nielsen. 2012b. Getting started with the glmmADMB package. Online at: *http://glmmadmb.r-forge.r-project.org/glmmADMB.html#x1-20131*

Gilchrist, TT, RJ Letcher, P Thomas, and KJ Fernie. 2014. Polybrominated diphenyl ethers and multiple stressors influence the reproduction of free-ranging tree swallows (*Tachycineta bicolor*) nesting at wastewater treatment plants. *Science of the Total Environment* 427:63-71.

R Core Team. 2013. R: a language and environment for statistical computing. R Foundation for Statistical Computing, Vienna, Austria. Online at: *http://www.R-project.org/.*

Skaug, H, D Fournier, A Nielsen, A Magnusson, and BM Bolker. 2013. Generalized linear mixed models using AD model builder. Version 0.7.7. Statistical package for R.

Table S1. Comparison of models used in supplemental analyses of performance ratios in Tree Swallows (using ∆AICc)^1^.

| Fixed effects | glmmADMB-NB-ZI^2^ | glmm-ADMB-HB^3^ | glmmADMB-HNZ^4^ |
| --- | --- | --- | --- |
| null model | 3.2 | 0.0 | 5.8 |
| performance category | 0.0 | 1.9 | 0.0 |
| PCB level | 5.2 | 1.1 | 7.7 |
| PCB level + category | 2.1 | 2.8 | 1.9 |
| PCB level * category | 4.2 | 3.0 | 3.2 |

^1^ models compared using delta Akaike’s Information Criterion adjusted for small sample size (∆AICc). The best-fit model is indicated by a 0.0

^2^ negative binomial, zero-inflated

^3^ hurdle model, binomial component

^4^ hurdle model, non-zero (count) component

Figure S1. Tree Swallow timing of breeding plotted against total PCB concentrations (from nestling tissue, except for Ontario, Canada data from [1], which reports egg concentrations). First egg dates for the Hudson River, NY study [2] are plotted in a separate panel (B) to expand the x-axis for the lower PCB sites plotted in panel A. Studies are grouped by symbols and abbreviations provided in the figure legend: Ontario, Canada [1]; Lake Calumet [3]; and Fraser River [4].

Figure S2. Tree Swallow nest construction data (number of feathers in the nest at the time of hatching, and mass of the nest at the time of hatching) plotted against total nestling PCB concentrations from the Hudson River, NY study [5].

Figure S3. Tree Swallow clutch size (# of eggs) plotted against total PCB concentrations (from nestling tissue, except for Ontario, Canada data from [1], which reports egg concentrations). The Hudson River and New Bedford studies are plotted in panel B so that the x-axis in panel A could be expanded. Studies are grouped by symbols and abbreviations provided in the figure legend: Great Lakes & St. Lawrence R [pers. comm. with C. Bishop and 6]; Fox R [7]; Kalamazoo R [8]; Fraser R [4]; Ontario, Canada [1]; Lake Calumet [3]; Hudson R, NY [2]; and Fox Hill, RI & New Bedford, MA [9].

Figure S4. Tree Swallow egg mass plotted against total nestling PCB concentrations from the Hudson River, NY [5] and Kalamazoo River, MI [8] studies.

Figure S5. Tree Swallow hatching success (estimated in various ways as specified in the legend) plotted against total PCB concentrations (from nestling tissue, except for Ontario, Canada data from [1], which reports egg concentrations). Studies are grouped by symbols and abbreviations provided in the figure legend: Fox R [7]; Great Lakes & St. Lawrence R [pers. comm. with C. Bishop and 6]; Housatonic R [10]; Fox Hill, RI & New Bedford, MA [9]; Kalamazoo R [8]; Ontario, Canada [1]; and Lake Calumet [3].

Figure S6. Percentage of Tree Swallow eggs and clutches containing dead embryos plotted against total PCB concentrations from nestling tissue reported in a study conducted in the Fox River drainage and Green Bay, Wisconsin [7].

Figure S7. Nestling Tree Swallow morphology (wing chord, body length, tarsus length) plotted against total PCB concentrations from nestling tissue. Wing chord data from the Hudson River, NY study [2] are plotted in panel B so that the x-axis in panel A could be expanded. Studies are grouped by symbols and abbreviations provided in the figure legend: Kalamazoo R [8] and Fraser R [4].

Figure S8. Nestling Tree Swallow body mass plotted against total PCB concentrations from nestling tissue. Studies are grouped by symbols and abbreviations provided in the figure legend: Great Lakes & St. Lawrence R [pers. comm. with C. Bishop and 6]; Lake Calumet [3]; Kalamazoo R [8]; and Fraser R [4].

Figure S9. Tree Swallow breeding success (estimated in various ways as specified in the legend) plotted against total PCB concentrations (from nestling tissue, except for Ontario, Canada data from [1], which reports egg concentrations). Breeding success data from the Hudson River, NY study [2] are plotted in panel B so that the x-axis in panel A could be expanded. Studies are grouped by symbols and abbreviations provided in the figure legend: Great Lakes & St. Lawrence R [pers. comm. with C. Bishop and 6]; Kalamazoo R [8]; Ontario, Canada [1]; Lake Calumet [3]; and Fraser R [4].

List of studies included in analyses (data plotted in figures above):

1 Gilchrist, T. T., Letcher, R. J., Thomas, P., Fernie, K. J. 2014 Polybrominated diphenyl ethers and multiple stressors influence the reproduction of free-ranging tree swallows (*Tachycineta bicolor*) nesting at wastewater treatment plants. *Science of the Total Environment*. **472**, 63-71. (http://dx.doi.org/10.1016/j.scitotenv.2013.10.090)

2 Secord, A. L., McCarty, J. P. Polychlorinated biphenyl contamination of tree swallows in the Upper Hudson River Valley, New York. Effects on breeding biology and implications for other bird species.: U. S. Fish and Wildlife Service Report 1997.

3 Soucek, D. J., Levengood, J. M., Gallo, S., Hill, W. R., Bordson, G. O., Talbott, J. L. 2013 Risk to birds in the Lake Calumet Region from contaminated emergent aquatic insects: Illinois Sustainable Technology Center Report, Prairie Research Institute.

4 Harris, M. L., Elliott, J. E. 2000 Reproductive success and chlorinated hydrocarbon contamination in tree swallows (*Tachycineta bicolor*) nesting along rivers receiving pulp and paper mill effluent discharges. *Environmental Pollution*. **110**, 307-320. (http://dx.doi.org/10.1016/S0269-7491(99)00296-1)

5 McCarty, J. P., Secord, A. L. 1999 Nest-building behavior in PCB-contaminated tree swallows. *The Auk*. 55-63.

6 Bishop, C. A., Mahony, N. A., Trudeau, S., Pettit, K. E. 1999 Reproductive success and biochemical effects in tree swallows (*Tachycineta bicolor*) exposed to chlorinated hydrocarbon contaminants in wetlands of the Great Lakes and St. Lawrence River Basin, USA and Canada. *Environmental Toxicology and Chemistry*. **18**, 263-271. (10.1002/etc.5620180224)

7 Custer, C. M., Custer, T. W., Allen, P. D., Stromborg, K. L., Melancon, M. J. 1998 Reproduction and environmental contamination in tree swallows nesting in the Fox River Drainage and Green Bay, Wisconsin, USA. *Environmental Toxicology and Chemistry*. **17**, 1786-1798. (10.1002/etc.5620170919)

8 Neigh, A. M., Zwiernik, M. J., Joldersma, C. A., Blankenship, A. L., Strause, K. D., Millsap, S. D., Newsted, J. L., Giesy, J. P. 2007 Reproductive success of passerines exposed to polychlorinated biphenyls through the terrestrial food web of the Kalamazoo River. *Ecotoxicology and Environmental Safety*. **66**, 107-118. (http://dx.doi.org/10.1016/j.ecoenv.2005.10.004)

9 Jayaraman, S., Nacci, D. E., Champlin, D. M., Pruell, R. J., Rocha, K. J., Custer, C. M., Custer, T. W., Cantwell, M. 2009 PCBs and DDE in Tree Swallow (*Tachycineta bicolor*) Eggs and nestlings from an estuarine PCB Superfund Site, New Bedford Harbor, MA, U.S.A. *Environmental Science and Technology*. **43**, 8387-8392. (10.1021/es900255v)

10 Custer, C. M., Custer, T. W., Dummer, P. M., Munney, K. L. 2003 Exposure and effects of chemical contaminants on tree swallows nesting along the Housatonic River, Berkshire County, Massachusetts, USA, 1998–2000. *Environmental Toxicology and Chemistry*. **22**, 1605-1621. (10.1002/etc.5620220725)
